# Unpacking the influence of sleep characteristics on the relationship between 24‐hour time‐use patterns and cognitive performance in older adults: an exploratory study using compositional data analysis

**DOI:** 10.1002/alz70860_098141

**Published:** 2025-12-23

**Authors:** Maddison L Mellow, Dorothea Dumuid, Hannah Keage, Frini Karayanidis, Ashleigh E Smith

**Affiliations:** ^1^ Alliance for Research in Exercise, Nutrition and Activity (ARENA) Research Centre, University of South Australia, Adelaide, SA, Australia; ^2^ Cognitive Ageing and Impairment Neurosciences Laboratory (CAIN), University of South Australia, Adelaide, SA, Australia; ^3^ Functional Neuroimaging Laboratory, University of Newcastle, Newcastle, NSW, Australia; ^4^ Healthy Minds Research Program, Hunter Medical Research Institute, Newcastle, NSW, Australia

## Abstract

**Background:**

The balance of sleep, physical activity, and sedentary behaviours in the 24‐hr day (i.e., time‐use composition) is associated with cognitive performance in older adults. However, it is unclear how factors such as sleep quality impact this relationship. This cross‐sectional study investigated whether sleep quality impacts the relationship between 24‐hr time‐use composition and cognitive performance in older adults without dementia.

**Method:**

Baseline data from the ACTIVate study (*n* = 368, mean age=65y) were used. Self‐reported daily activity and sleep patterns, collected using the Multimedia Activity Recall for Children and Adults, were expressed as a 24‐hour time‐use composition (min/day in sleep, sport/exercise, chores, household administration, quiet time, screen time, social and self‐care domains). The Pittsburgh Sleep Quality Index total score was used to classify participants as having ‘good’ (<5; *n* = 151) or ‘poor’ (≥5; *n* = 217) sleep quality. Memory, executive function and processing speed were measured using the Cambridge Neuropsychological Test Automated Battery. Within ‘good’ and ‘poor’ sleep quality subgroups, associations between time‐use composition (expressed as isometric log‐ratios) and cognitive outcomes were tested using multiple linear regression adjusted for age, sex, and education. Post‐hoc plotting explored how reallocations of time between time‐use domains were associated with cognitive outcomes within sleep quality sub‐groups.

**Result:**

Compared to good quality sleepers, poor quality sleepers spent more time in chores and quiet time, and less time in sport/exercise and household administration domains. Time‐use composition was associated with memory in poor quality sleepers (*p* = 0.04) but not good quality sleepers (*p* = 0.26), and there were no associations with executive function or processing speed for either group. Reallocating time to sport/exercise at the expense of any other time‐use domain was beneficial for memory in poor sleepers, with the greatest benefit observed when time was reallocated from sleep or quiet time. Conversely in good sleepers, increasing time in sport/exercise was only beneficial for memory if reallocated directly from screen time.

**Conclusion:**

Subjective sleep quality altered the association between 24‐hr time use and memory performance, whereby poor quality sleepers benefited more from time in sport/exercise. Personalised 24‐hr time‐use interventions may be more beneficial if tailored based on current sleep quality.

